# 1276. Mechanical, Microbiological, and Inflammatory Factors May Impact 1-Year Revision Rates after Arthroplasty Resection for Periprosthetic Joint Infection

**DOI:** 10.1093/ofid/ofad500.1116

**Published:** 2023-11-27

**Authors:** Rawabi Aljadani, Hilal Maradit Kremers, Martha Carvour

**Affiliations:** University of Iowa, Iowa City, Iowa; Mayo Clinic, Rochester, Minnesota; University of Iowa Hospitals and Clinics, Iowa City, Iowa

## Abstract

**Background:**

Periprosthetic joint infection (PJI) is often managed using a combination of medical and surgical treatments, including irrigation and debridement or joint resection, spacer placement, and then revision to reimplant the arthroplasty components. The timing between surgical stages may vary, and little information exists about the clinical and microbiological factors impacting these temporal relationships.

**Methods:**

A single center cohort study was conducted. The cohort included all cases of PJI for which resection was performed at the University of Iowa Health Care (UIHCs) between January 1, 2009, and December 31, 2016. Using a 1-year follow-up period, we constructed Kaplan-Meier estimates of the time to revision after resection—overall and then stratified by joint location (knee vs. hip), organism identification (known vs unknown), and C-reactive protein level (CRP >10 mg/L vs. CRP ≤10 mg/L). We then used Cox proportional hazard modeling to identify factors associated with revision within 1 year.

**Results:**

Among a total of 109 cases of prosthetic joint resections (n = 67 knee, n = 42 hip), 56.9% underwent revision surgery during the follow-up period (n = 46 knee, n= 16 hip). Most revisions occurred between 2.5 and 3.0 months after resection. Knee arthroplasties were significantly more likely to undergo revision during the study period (hazard ratio, HR = 2.53, 95% confidence interval, CI: 1.38, 4.94) compared to hip arthroplasties. Identification of one or more organism involved in the PJI was also associated with revision during the study window (HR = 3.56, 95% CI: 1.94, 6.47), whereas elevated CRP was associated with lower rates of revision during the study period (HR = 0.21, 95% CI: 0.09, 0.46 for CRP >10 mg/L compared to CRP ≤10 mg/L).

**Figure 1.** Kaplan-Meier curves for revision within 1-year after resection for the (A) cohort overall and then stratified by (B) joint location (hip vs knee), (C) organism identification (known vs unknown), and (D) C-reactive protein level (CRP >10 mg/L vs. CRP ≤10 mg/L).

**Figure:**
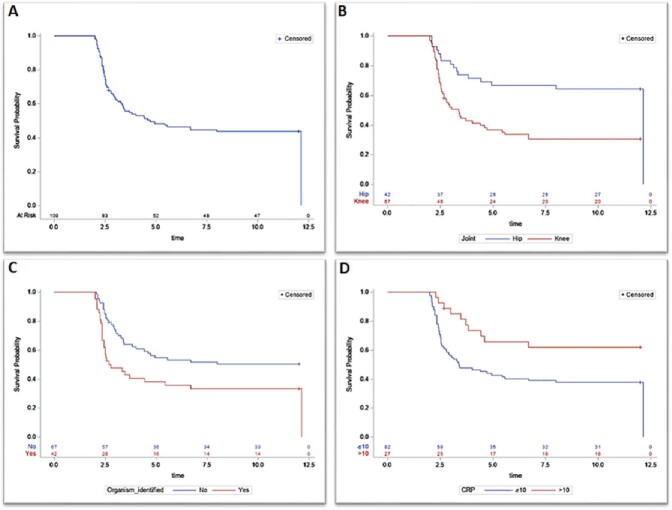

**Conclusion:**

For patients with surgically managed PJIs, joint location, organism identification, and lower CRP level were all associated with revision procedures within 1-year after resection. These findings may reflect mechanical and microbiological factors influencing the decision to proceed with revision. The variable length between stages—and thus, the variable time during which a spacer is retained—may be important to measure in future observational and clinical studies examining PJI outcomes.

**Disclosures:**

**All Authors**: No reported disclosures

